# Minimally Invasive Saliva Testing to Monitor Norovirus Infection in Community Settings

**DOI:** 10.1093/infdis/jiy638

**Published:** 2018-12-05

**Authors:** Nora Pisanic, Sarah-Blythe Ballard, Fabiola D Colquechagua, Ruthly François, Natalie Exum, Pablo Peñataro Yori, Kellogg J Schwab, Douglas A Granger, Barbara Detrick, Maribel Paredes Olortegui, Holger Mayta, Gerardo J Sánchez, Robert H Gilman, Christopher D Heaney, Jan Vinjé, Margaret N Kosek

**Affiliations:** 1 Department of Environmental Health and Engineering, Johns Hopkins Bloomberg School of Public Health, Baltimore; 2 Departments of International Health, Johns Hopkins Bloomberg School of Public Health, Baltimore; 3 Department of Preventive Medicine and Biostatistics, Uniformed Services University of the Health Sciences, Bethesda, Maryland; 4 Infectious Diseases Research Laboratory, Department of Cellular and Molecular Sciences, Universidad Peruana Cayetano Heredia, Lima, Peru; 5 Institute for Interdisciplinary Salivary Bioscience, University of California, Irvine; 6 Department of Pediatrics, Johns Hopkins University School of Medicine, Baltimore, Maryland; 7 Department of Acute and Chronic Care, Johns Hopkins University School of Nursing, Baltimore, Maryland; 8 Department of Population, Family, and Reproductive Health, Johns Hopkins Bloomberg School of Public Health, Baltimore, Maryland; 9 Department of Pathology, Johns Hopkins University School of Medicine, Baltimore, Maryland; 10 Asociación Benéfica Prisma, Iquitos, Perú; 11 Department of Epidemiology, Johns Hopkins Bloomberg School of Public Health, Baltimore, Maryland; 12 National Calicivirus Laboratory, Centers for Disease Control and Prevention, Atlanta, Georgia

**Keywords:** norovirus, saliva, multiplex immunoassay, noninvasive diagnostics, MAL-ED

## Abstract

**Background:**

Norovirus is a leading cause of acute gastroenteritis worldwide. Routine norovirus diagnosis requires stool collection. The goal of this study was to develop and validate a noninvasive method to diagnose norovirus to complement stool diagnostics and to facilitate studies on transmission.

**Methods:**

A multiplex immunoassay to measure salivary immunoglobulin G (IgG) responses to 5 common norovirus genotypes (GI.1, GII.2, GII.4, GII.6, and GII.17) was developed. The assay was validated using acute and convalescent saliva samples collected from Peruvian children <5 years of age with polymerase chain reaction (PCR)–diagnosed norovirus infections (n = 175) and controls (n = 32). The assay sensitivity and specificity were calculated to determine infection status based on fold rise of salivary norovirus genotype-specific IgG using norovirus genotype from stool as reference.

**Results:**

The salivary assay detected recent norovirus infections and correctly assigned the infecting genotype. Sensitivity was 71% and specificity was 96% across the evaluated genotypes compared to PCR-diagnosed norovirus infection.

**Conclusions:**

This saliva-based assay will be a useful tool to monitor norovirus transmission in high-risk settings such as daycare centers or hospitals. Cross-reactivity is limited between the tested genotypes, which represent the most commonly circulating genotypes.

## Teaser

This study describes the performance of a multiplex immunoassay to measure salivary immunoglobulin G responses to multiple norovirus genotypes. The assay diagnosed recent norovirus infections at the genotype level with 71% sensitivity and 96% specificity compared to stool-based polymerase chain reaction diagnosis.

Norovirus causes an estimated 19–21 million cases of acute gastroenteritis (AGE) in the United States each year, leading to 56000–71000 hospitalizations and 570–800 deaths, mostly among young children and older adults. Norovirus is the leading cause of severe AGE among medical care–seeking US children <5 years of age, and a principal cause of AGE outbreaks on cruise ships and in preschools, hospitals, and long-term care facilities [1–5].

The current gold standard for norovirus laboratory diagnosis involves viral nucleic acid extraction from fecal samples and quantitative reverse-transcription polymerase chain reaction (RT-qPCR) for genogroup I (GI) and GII, followed by conventional RT-PCR and sequencing of a partial region of the capsid and polymerase genes and subsequent sequence-based genotyping of the virus [6–8].

Stool sample collection and testing is a feasible method in clinical settings where patients with severe AGE present seeking care; however, patients and family members are inconvenienced by stool collection and handling and often do not provide appropriate samples [9]. An alternative approach to diagnose infection is measuring pathogen-specific antibody levels in blood [10]. But blood collection is invasive, usually requires clinically trained personnel, and has practical constraints among young children. More convenient testing methods would greatly increase the coverage of diagnostic testing in epidemiologic studies, outbreak investigations, and disease surveillance.

Saliva collection is minimally invasive, does not require clinical personnel, and can be implemented easily in any setting including remote areas and community-based settings [11–13]. Saliva harbors pathogen-specific immunoglobulin A (IgA) and immunoglobulin G (IgG) antibodies, and immunoassays based on saliva can detect pathogen exposure with similar sensitivity and specificity as blood-based immunoassays in adults [13]. Several studies have shown an increase in norovirus-specific IgG and IgA in saliva postchallenge with Norwalk virus (GI.1) and Snow Mountain virus (GII.2) [14–17]. Griffin et al demonstrated that multiplex immunoassays for salivary antibodies to a variety of waterborne pathogens including 3 norovirus genotypes (GI.1, GII.4, and GII.9) could detect a GII.4 infection in an adult volunteer and that salivary immunoassays to detect IgG and IgA against Norwalk virus could correctly identify 3 infected and 4 noninfected volunteers in a challenge study [16, 17]. However, salivary norovirus genotype-specific IgG responses among children <5 years of age with PCR-diagnosed norovirus infections have not been described previously.Our goal was to develop and validate a multiplex norovirus assay to measure antibody responses in saliva to 5 common norovirus genotypes (GI.1, GII.2, GII.4, GII.6, and GII.17) and to describe the salivary IgG response to those genotypes among children with stool-diagnosed norovirus infection and controls. We aimed to (1) investigate the change in norovirus genotype-specific IgG in saliva between the acute and convalescent phase of norovirus infection and (2) determine the salivary norovirus IgG assay’s sensitivity and specificity to diagnose norovirus infection at the genotype level compared to molecular diagnosis by RT-PCR of the infecting genotype in stool in order to provide a practical tool for the evaluation of norovirus outbreaks and study of transmission.

## MATERIALS AND METHODS

### Study Design and Sample Collection

For this study, data and samples were collected from children <5 years of age who participated in 2 research studies in Peru. One study was a case-control study designed to evaluate the etiology of medically attended AGE in children <5 years of age following nationwide rotavirus vaccine implementation in Peru. Children were enrolled at the Instituto Nacional de Salud Del Niño in Lima, Peru, from October 2013 through May 2015. Children presenting with symptoms of AGE were defined as cases; children seeking care unrelated to AGE and without a diarrheal episode within the past 30 days were defined as controls. AGE was defined according to the World Health Organization initiative to estimate the global burden of foodborne diseases [18]. Caregivers provided demographic and symptom information and study personnel collected a stool and a saliva sample (SalivaBio Children’s Swab, Salimetrics). Children with acute norovirus infections (defined as a stool sample positive for norovirus GI or GII by RT-PCR) were visited by study personnel at least 21 days later to collect a convalescent phase saliva sample. Timing for convalescent phase saliva collection was informed by a previous study describing that 83% of infected individuals had a ≥4-fold increase in salivary IgG 21 days post–norovirus challenge and that salivary IgG continued to rise through 21 days postchallenge [19]. Children enrolled as controls provided 1 saliva sample. Preliminary analyses of stool samples from children enrolled into this study showed that 36.2% of children enrolled as cases and 12.4% of children enrolled as controls had norovirus-positive stools.

Because this study was not designed to compare the change in antibody against norovirus between cases and controls (ie, no second saliva sample was collected among controls), additional samples collected among children who participated in the Etiology, Risk Factors, and Interactions of Enteric Infections and Malnutrition and the Consequences for Child Health and Development (MAL-ED) study in Iquitos, Peru, were included in the analysis. The MAL-ED birth cohort study has been described in detail previously [20–23]. A subset of children who participated in the MAL-ED study in Iquitos provided monthly and diarrhea-triggered stool samples and weekly saliva samples (Oracol saliva swab, Malvern Medical Development) between May and September 2015. Children in the MAL-ED birth cohort were approximately 4–5 years old during the sample collection period. To match the Lima study design, 2 saliva samples from each child that were collected during the acute and convalescent phase of infection or the closest available sampling time points were included in the analysis. More specifically, from children with norovirus infection during the 4-month sampling period, the last saliva sample before a stool-confirmed norovirus infection and a second saliva sample collected 4 weeks later or the closest available were selected (cases). For children without norovirus infection during the sampling period (defined as monthly stool samples norovirus GI and GII negative by RT-qPCR), 2 saliva samples that were collected 4 weeks apart were selected randomly (controls). This study was approved by Asociación Benéfica Prisma and Universidad Peruana Cayetano Heredia (Lima, Peru) and the Johns Hopkins Bloomberg School of Public Health Institutional Review Board (Baltimore, Maryland).

### Norovirus Genotyping From Stool Samples

RNA was extracted from a 10% wt/vol suspension of stool in phosphate-buffered saline using the Qiagen QIAmp viral RNA kit according to the manufacturer’s instructions and tested for norovirus GI and GII by RT-qPCR [24]. Samples were considered positive if the cycle threshold for the sample was ≤37 for norovirus GI and ≤39 for GII. For positive samples, a partial region of the capsid gene (region C) was amplified. Amplicons were then purified and sequenced. The generated sequences were genotyped using the online NoroNet sequence typing tool [25, 26] or using http://norovirus.phiresearchlab.org/.

### Multiplex Immunoassay for Norovirus Genotype-Specific IgG in Saliva

Virus-like particles (VLPs) for 5 norovirus genotypes (GI.1, GII.2, GII.4, GII.6, and GII.17) were kindly provided by Dr Robert Atmar and covalently coupled to 5 magnetic microparticle sets (MagPlex microspheres, Luminex) as described previously [13]. Coupling of VLPs to beads was confirmed using monoclonal mouse antibodies against GI.1 and GII.4 (Maine Biotechnology Services) followed by R-phycoerythrin (PE)–labeled anti-mouse antibody and convalescent sera from patients with known GI.1 and GII.4 infections followed by PE-labeled anti-human IgG antibody (Jackson ImmunoResearch Laboratories) and revealed a fluorescent signal of >20000 mean fluorescence intensity (MFI).

Saliva samples were centrifuged (5 minutes at 10000*g*, 20°C), and 10 μL of saliva supernatant was added to 40 μL of assay buffer (phosphate-buffered saline with 0.05% Tween20 and 1% bovine serum albumin) containing 2000 beads of each bead set (GI.1, GII.2, GII.4, GII.6, and GII.17 VLPs) per microplate well. The plate was covered and incubated at room temperature for 1 hour on a plate shaker at 500 rpm. Beads were washed 3 times, 50 μL of PE-labeled anti-human IgG diluted 1:100 in assay buffer was added, and the plate was incubated for 1 hour on a plate shaker at 500 rpm. Beads were again washed and were suspended in 100 μL of sheath fluid (Luminex), and the fluorescence signal was measured on a Bio-Plex 200 instrument (Bio-Rad). A subset (~10%) of saliva samples was tested in duplicate to determine intra-assay variability, and 2 blanks (assay buffer) were included on each plate for background subtraction.

### Statistical Analysis

Receiver operating characteristic (ROC) analyses based on logistic regression models were used to determine if (1) the fold rise of the salivary norovirus genotype-specific IgG response, (2) the difference between acute and convalescent IgG response, or (3) the difference between acute and convalescent IgG response divided by the time in days since the first sample collection (slope) would correlate best (highest area under the curve [AUC]) with infection status. Logistic regression models were used to estimate the association between the fold rise of norovirus genotype-specific IgG MFI in convalescent (or second) samples compared to acute phase (or first) saliva samples and RT-PCR–confirmed norovirus genotype infection status. Models were adjusted for all measured salivary IgG responses in the same model to account for potential cross-reactivity of salivary IgG to other genotypes included in the assay.

Several threshold definitions to determine infection status were explored and the sensitivity and specificity compared to the stool-diagnosed norovirus genotype were calculated. This included defining infection with genotypes GI (GI.2, GI.3, and GI.5 were combined as GI), GII.4, GII.6, or GII.17 as (1) a fold rise in genotype-specific IgG that is larger than the 95th percentile among controls, and (2) a >2-fold and (3) a >3-fold rise in salivary IgG against the norovirus genotype that elicited the highest immune response.

## RESULTS

Among the Lima cohort, 386 saliva samples from 235 children were collected. Children enrolled in Lima with stool-confirmed norovirus infection (cases; n = 151) provided a stool and saliva sample at their first visit (acute phase) and a second (convalescent phase) saliva sample on average 1 month later (median, 32 [range, 10–195] days). Children without norovirus infection (controls; n = 84) provided a single saliva sample. The MAL-ED cohort of 77 children in Iquitos, Peru, provided 1004 saliva samples and 333 stool samples. Children with at least 1 norovirus infection (stool sample positive for norovirus GI or GII by RT-qPCR) were defined as cases (41/77 [53%]); children without stool-confirmed norovirus infection over the 4-month study period were defined as controls (36/77 [47%]).

### Sample Selection

Among the Lima cohort, controls (n = 84) were excluded from the analysis because no second saliva sample was available and therefore the fold rise in genotype-specific IgG could not be calculated. Additionally, 2 cases were excluded because of insufficient saliva volume (<10 μL). Similarly, among the MAL-ED cohort, 20 children were excluded from the analysis because no saliva sample was available either before or at least 10 days after the stool-diagnosed norovirus infection (16 cases) or because only 1 saliva sample was available (4 controls). The sample selection process and resulting sample numbers used in the following analysis are described in Table 1. The mean age of children with stool-confirmed norovirus infection (Lima and MAL-ED cases combined, n = 175) was 23 months (range, 2–59 months) and of children without norovirus infection (MAL-ED controls, n = 32) was 44 months (range, 39–49 months). A slightly higher mean age among MAL-ED controls was expected due to the 2 different study designs. Children aged <5 years were eligible for enrollment into the Lima study cohort, whereas the MAL-ED birth cohort children were 3–5 years old during the sample collection period. For the following analyses, cases from both cohorts and controls from the MAL-ED cohort were combined.

**Table 1.** Sample Selection Criteria for Inclusion in Analysis of Children With and Without Norovirus Infection in Lima and Iquitos, Peru, 2014–2015

Sample	Lima Cohort			Iquitos Cohort			Total
	Cases	Controls	Combined	Cases	Controls	Combined	
Samples collected, No.							
Children	151	84	235	41	36	77	312
Saliva samples	302	84	386	529	475	1004	1390
Stool samples	151	84	235	180	153	333	568
Samples included in analysis^a^							
Children	149	none	149	25^b^	32	57	206
Saliva samples	298	none	298	52	64	116	414
Stool samples	149	none	149	26	32	58	207

^a^Inclusion criteria for analysis: ≥2 saliva samples with ≥10 µL volume available per child; saliva samples collected ≥10 days apart. Cases: first saliva sample collected ≤3 days prior to norovirus-positive stool sample. Controls: first saliva sample collected ≤3 days prior to norovirus-negative stool sample.

^b^One child contributed 4 saliva samples and 2 norovirus-positive stools (different genotypes) collected >1 month apart.

### Norovirus Genotype Distribution

**Figure 1.**
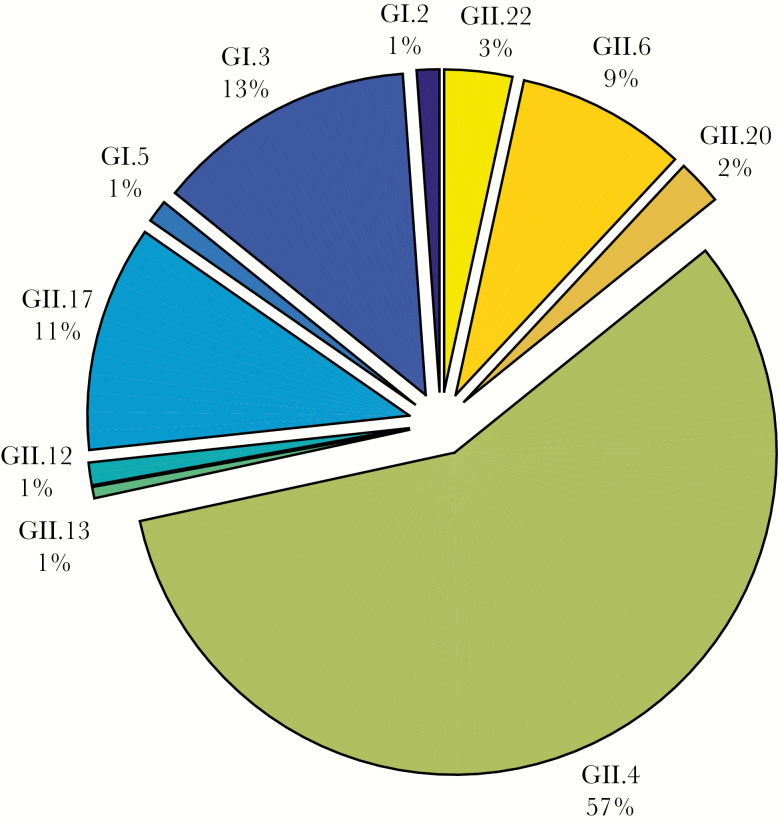
Norovirus genotype distribution among children presenting with acute gastroenteritis (n = 149) at a children’s hospital in Lima, and among children in Iquitos (n = 25), Peru, 2014–2015. One child was infected with 2 genotypes (GI.3 and GII.22).

The norovirus genotype distribution among children with norovirus infection is presented in Figure 1. Norovirus GII.4 was the predominant genotype (n = 101 [57%]), followed by genotypes GI.3 (n = 23 [13%]), GII.17 (n = 20 [11%]), and GII.6 (n = 15 [9%]).

### Norovirus Genotype-Specific IgG Response in Saliva

The median anti-GI.1, anti-GII.4, anti-GII.6, and anti-GII.17 IgG MFI was higher in convalescent samples compared to acute samples among children with GI, GII.4, GII.6, and GII.17 infections, respectively (Table 2). The median fold rise of anti-GI.1, anti-GII.4, anti-GII.6, and anti-GII.17 IgG of convalescent to acute samples was also higher among children infected with the respective genotype (27 GI, 101 GII.4, 15 GII.6, and 20 GII.17 cases) compared to children infected with other genotypes and controls, suggesting that norovirus infections elicit a genotype-specific IgG response that can be measured in saliva. However, the standard deviation of the salivary IgG response between children with the same infecting genotype and among controls was high. We did not observe associations between the fold rise in salivary anti-norovirus IgG among GI, GII.4, GII.6, and GII.17 cases and age.

**Table 2.** Norovirus Genotype-Specific Salivary Immunoglobulin G Response in Acute and Convalescent Saliva Samples From Children With GI, GII.4, GII.6, and GII.17 Infections Compared to Children With Other Norovirus Genotype Infections and Controls

Assay Analyte	Sample Group	No.	First (Acute) Sample, MFI	Second (Convalescent) Sample, MFI	Days Since First Sample	Fold Rise (Convalescent/ Acute)
Anti-GI.1 IgG	GI cases	27	293 (941)	1605 (5193)	29 (30)	4 (18)
	Non-GI cases	148	61 (1357)	38 (1671)	31 (32)	1 (11)
	Controls	32	505 (2003)	560 (821)	28 (14)	1 (5)
Anti-GII.2 IgG	GII.2 cases	none	NA	NA	NA	NA
	Non-GII.2 cases	175	68 (923)	135 (2052)	30 (31)	2 (22)
	Controls	32	878 (1122)	506 (1924)	28 (14)	1 (1)
Anti-GII.4 IgG	GII.4 cases	101	67 (817)	2139 (4858)	32 (34)	30 (134)
	Non-GII.4 cases	74	577 (2591)	713 (2934)	29 (26)	1 (57)
	Controls	32	1641 (1945)	1706 (3042)	28 (14)	1 (2)
Anti-GII.6 IgG	GII.6 cases	15	46 (456)	1447 (2688)	26 (34)	15 (191)
	Non-GII.6 cases	160	327 (3745)	715 (4312)	31 (31)	1 (28)
	Controls	32	3542 (4002)	2796 (4388)	28 (14)	1 (2)
Anti-GII.17 IgG	GII.17 cases	20	115 (490)	281 (1015)	34 (18)	3 (34)
	Non-GII.17 cases	155	59 (1436)	82 (1958)	30 (33)	1 (13)
	Controls	32	596 (1494)	511 (1513)	28 (14)	1 (3)

Data are presented as median (standard deviation). Data from 175 cases with norovirus infection (149 from Lima cohort and 26 from the Etiology, Risk Factors, and Interactions of Enteric Infections and Malnutrition and the Consequences for Child Health and Development [MAL-ED] cohort) and from 32 controls (MAL-ED cohort only) were included in this analysis.

Abbreviations: IgG, immunoglobulin G; MFI, mean fluorescence intensity; NA, not applicable.

### Intra-assay Variability

The average intra-assay variability (coefficient of variation [CV%]) to detect anti-GI.1, GII.2, GII.4, GII.6, and GII.17 IgG was 3.5%, 4.2%, 2.3%, 3.4%, and 4.8%, respectively. These values represent the average CV% of 30 saliva samples (~7.5% of all samples) that were each measured in duplicate. The observed variability lies below assay precision criteria (±15% CV) recommended for commercial assays [27].

### ROC Curves

**Figure 2.**
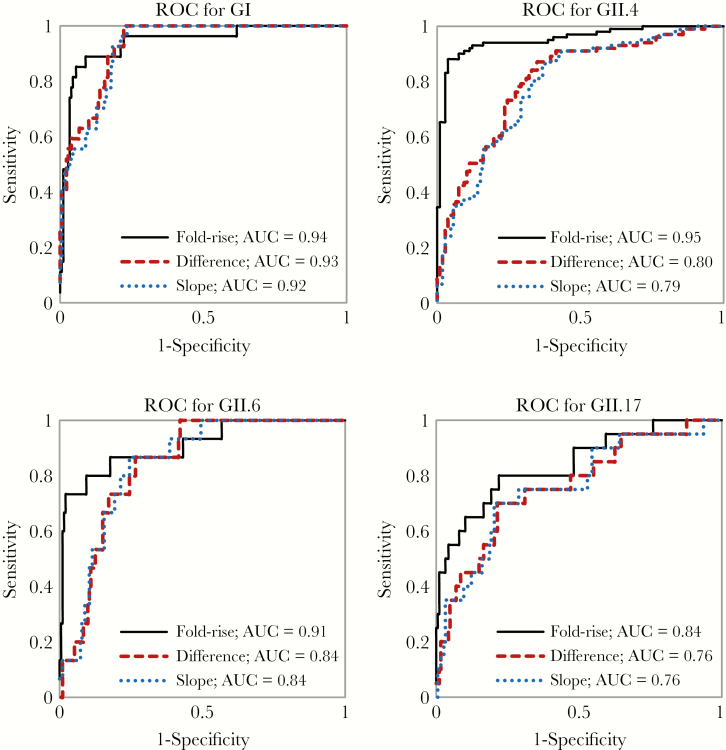
Receiver operating characteristic (ROC) with corresponding area under the curve (AUC) of the multiplex assay for salivary norovirus genotype-specific immunoglobulin G to diagnose GI, GII.4, GII.6, and GII.17 infections among children <5 years of age in Lima and Iquitos, Peru, 2014–2015. Data from 175 cases with norovirus infection (149 from Lima cohort and 26 from Etiology, Risk Factors, and Interactions of Enteric Infections and Malnutrition and the Consequences for Child Health and Development [MAL-ED] cohort) and from 32 controls (MAL-ED cohort only) were included in this analysis.

Figure 2 shows the ROC curves based on logistic regression models to estimate the association between (1) fold rise of GI-, GII.2-, GII.4-, GII.6-, and GII.17-specific IgG MFI in convalescent compared to acute samples; (2) difference in genotype-specific IgG MFI; and (3) difference in genotype-specific IgG MFI divided days since first sample collection and stool-diagnosed infection outcome.

The fold rise in salivary norovirus IgG yielded the highest AUC for each outcome (GI, GII.4, GII.6, and GII.17 infection by RT-PCR from stool) and was therefore selected for further assay validation.

### Association Between Fold Rise in Salivary Genotype-Specific IgG MFI and PCR-Diagnosed Genotype

We estimated associations between fold rise in genotype-specific IgG MFI in convalescent (or second samples among controls) compared to acute samples (or first samples among controls) and GI, GII.4, GII.6, and GII.17 infection status (Table 3). Odds ratios in Table 3 compare the fold rise in genotype-specific IgG among cases with one genotype infection to cases with different genotype infections and to controls.

The odds of infection with the respective genotype increase 24- to 91-fold for every log_10_ unit increase in the genotype-specific IgG MFI fold rise variable when all genotype-specific IgG MFI responses (fold rise) represented in the multiplex assay were included in the model. However, a rise in anti-GII.2 IgG was also associated with higher odds for GI infection (Table 3), suggesting cross-reactivity between salivary anti-GI IgG and GII.2 VLPs used in this assay.

**Table 3.** Association Between Fold Rise of Genotype-Specific Immunoglobulin G Mean Fluorescence Intensity to 5 Norovirus Genotypes Following Infection and Reverse-Transcription Polymerase Chain Reaction–Diagnosed Genotype Among Children <5 Years of Age in Lima and Iquitos, Peru, 2014–2015

Norovirus Genotype Infection Status	No.	No. Positive	OR (95% CI)	*P* Value
GI	207	27		
Fold rise in salivary anti-GI IgG			56.2 (11.1–283.9)	**< .001**
Fold rise in salivary anti-GII.2 IgG			22.3 (1–488.6)	.049
Fold rise in salivary anti-GII.4 IgG			0.1 (0–.4)	.002
Fold rise in salivary anti-GII.6 IgG			0.6 (.2–2.7)	.538
Fold rise in salivary anti-GII.17 IgG			0.0 (.0–.6)	.028
GII.4	207	101		
Fold rise in salivary anti-GI IgG			0.6 (.2–1.4)	.220
Fold rise in salivary anti-GII.2 IgG			0.1 (0–1.3)	.080
Fold rise in salivary anti-GII.4 IgG			90.5 (24.2–338.6)	**< .001**
Fold rise in salivary anti-GII.6 IgG			0.1 (0–.2)	< .001
Fold rise in salivary anti-GII.17 IgG			4.8 (.4–56.9)	.213
GII.6	207	15		
Fold rise in salivary anti-GI IgG			0.5 (.1–2.2)	.375
Fold rise in salivary anti-GII.2 IgG			7.9 (.4–143.8)	.164
Fold rise in salivary anti-GII.4 IgG			0.2 (.1–.5)	.002
Fold rise in salivary anti-GII.6 IgG			24.3 (6.3–94.1)	**< .001**
Fold rise in salivary anti-GII.17 IgG			0.1 (0–1.7)	.1
GII.17	207	20		
Fold rise in salivary anti-GI IgG			0.2 (.1–.7)	.008
Fold rise in salivary anti-GII.2 IgG			0.2 (0–1.9)	.172
Fold rise in salivary anti-GII.4 IgG			0.2 (.1–.5)	< .001
Fold rise in salivary anti-GII.6 IgG			1.9 (.7–5.1)	.178
Fold rise in salivary anti-GII.17 IgG			54.6 (5.4–552.6)	**< .001**

Data from 175 cases with norovirus infection (149 from Lima cohort and 26 from the Etiology, Risk Factors, and Interactions of Enteric Infections and Malnutrition and the Consequences for Child Health and Development [MAL-ED] cohort) and from 32 controls (MAL-ED cohort only) were included in this analysis. ORs and 95% CIs are derived from logistic regression models adjusting for each of the independent variables within the same model. Independent variables: log_10_ of fold rise in genotype-specific IgG mean fluorescence intensity of convalescent/second to the acute/first saliva samples. Dependent variables for reverse-transcription polymerase chain reaction–diagnosed negative/positive norovirus GI, GII.4, GII.6, and GII.17 infection status were coded as 0/1.

Abbreviations: CI, confidence interval; IgG, immunoglobulin G; OR, odds ratio.

### Sensitivity and Specificity of the Salivary Norovirus Genotype IgG Assay Compared to RT-PCR Diagnosis From Stool

Multiple threshold definitions to discriminate cases from noncases and controls were explored and assay sensitivity and specificity were calculated using the RT-PCR–diagnosed norovirus genotype as reference. Thresholds explored to discriminate children with norovirus at the genotype level and without infection included (1) a fold rise in salivary norovirus genotype-specific IgG larger than the 95th percentile of the fold rise among controls; (2) if largest fold rise is >2-fold; and (3) if largest fold rise is >3-fold (Table 4).

**Table 4.** Sensitivity and Specificity of the Salivary Norovirus Genotype-Specific Immunoglobulin G Assay Compared to Reverse-Transcription Polymerase Chain Reaction–Diagnosed Norovirus Genotype Outcome

Genotype^a^	No.	Sensitivity, no./No. (%)	No.	Specificity, no./No. (%)
(1) Infecting genotype defined as a fold rise that is larger than the 95th percentile of fold rise among controls				
GI	27	8/27 (30)	180	171/180 (95)
GII.4	101	83/101 (82)	106	85/106 (80)
GII.6	15	13/15 (87)	192	130/192 (68)
GII.17	20	11/20 (55)	187	136/187 (73)
Weighted mean	163	115/163 (71)		76%
(2) Infecting genotype defined as genotype with largest fold rise if >2				
GI	27	16/27 (62)	180	177/180 (98)
GII.4	101	86/101 (85)	106	97/106 (92)
GII.6	15	11/15 (73)	192	179/192 (93)
GII.17	20	3/20 (15)	187	185/187 (99)
Weighted mean	163	116/163 (71)		96%
(3) Infecting genotype defined as genotype with largest fold rise if >3				
GI	27	12/27 (44)	180	177/180 (98)
GII.4	101	82/101 (81)	106	99/106 (93)
GII.6	15	11/15 (73)	192	182/192 (95)
GII.17	20	3/20 (15)	187	185/187 (99)
Weighted mean	163	108/163 (66)		97%

Data from 175 cases with norovirus infection (149 from Lima cohort and 26 from the Etiology, Risk Factors, and Interactions of Enteric Infections and Malnutrition and the Consequences for Child Health and Development [MAL-ED] cohort) and from 32 controls (MAL-ED cohort only) were included in this analysis. Cases were defined as (1) fold rise of convalescent to acute anti-norovirus genotype-specific immunoglobulin G (IgG) mean fluorescence intensity >95th percentile of the fold rise among controls; and (2) a >2-fold and (3) a >3-fold rise in salivary IgG against the norovirus genotype that elicited the highest immune response.

^a^Polymerase chain reaction–confirmed genotype from stool sample.

Case definitions (1) and (2) resulted in the highest sensitivity; however, using case definition 1, the specificity to diagnose GII.2, GII.6, and GII.17 infections was low (57%–76%). Case definition 2 resulted in the overall highest sensitivity (71%) and specificity (96%).

## DISCUSSION

We developed a multiplex assay to measure IgG responses to 5 norovirus genotypes in saliva. The assay was validated using acute and convalescent phase saliva samples collected from 175 children with confirmed norovirus infections and from 32 children without recent infection. The average sensitivity and specificity of the assay was 71% and 96%, respectively, compared with PCR diagnosis from stool.

The median convalescent phase norovirus genotype-specific IgG MFI was similar among children with or without a norovirus-positive stool, suggesting that most “controls” have been exposed to norovirus in the past. This finding is consistent with epidemiological studies that have demonstrated that children in Peru experience at least 1 GII infection by the age of 12 months and 1 GI infection by 24 months [20]. Hence, in highly endemic settings it is not feasible to apply anti-norovirus IgG MFI cutoffs to discriminate “seropositive” from “seronegative” samples based on a single time point, as most children have experienced multiple norovirus infections by the time they reach 5 years of age. It may, however, be possible to design a salivary norovirus assay to measure the acute phase IgA response in a single saliva sample [28]. The magnitude of the anti-norovirus IgA response may vary by child age and will vary by time since infection. In this highly endemic study setting, we focused only on the salivary IgG response, because we expected that a large proportion of children would experience a secondary immune response reflected by a rise in anti-norovirus IgG rather than a primary immune response reflected by a rise in anti-norovirus IgA.

We explored multiple cutoff definitions for immunoconversion as a proxy of previous infection and calculated the corresponding assay sensitivity and specificity to correctly identify the infecting norovirus genotype compared to PCR-based diagnosis in stool. The salivary assay sensitivity and specificity were highest for GII.4 and GII.6 infections using varying fold rise definitions as cutoff. The average time between acute and convalescent sample collection was highest among GI cases. Seven of the 11 GI cases that were misclassified by the salivary assay provided their convalescent sample on average 48 days (range, 44–90 days) after their acute sample was collected and may have been exposed to other genotypes during this time period, which may explain the lower sensitivity to detect GI cases. Most GII.17 cases that were misclassified by the salivary assay (13/17) showed a >3-fold rise in IgG against GII.2 (2/13 [15%]), GII.4 (2/13 [15%]), or GII.6 (9/13 [69%]), suggesting cross-reactivity between salivary anti-GII.17 IgG and GII.2, GII.4, and GII.6 VLPs.

Limitations to this study included the following. First, we collected 1 saliva sample from children in the Lima control group and could therefore not compare the assay performance between all cases and controls. The high number of cases, particularly of GII.4 cases, likely resulted in the negative associations observed between a rise in, for example, anti-GII.4 IgG and decreased odds of infection with GI. A higher number of controls and more equally distributed genotypes among cases might have attenuated these statistically significant negative associations (odds ratios <1). The reported assay sensitivity and specificity describe the assay performance to detect the correct genotype among, for example, children with GII.4 infections compared to children with other genotype infections and a comparatively small number of controls (n = 32). Second, 12 children experienced GII.12, GII.13, GII.20, or GII.22 infections. Samples from those children were included in the analysis (as non-GI, non-GII.4, etc, cases) and thus contributed to the specificity of the assay, but VLPs for those genotypes were not available and we therefore could not include those genotypes into the multiplex assay. Two children who were included in the analysis experienced coinfections with GI and GII, which likely decreased the assay performance as the assay interpretation only allowed for 1 outcome. Third, we had a high number of GII.4 cases (n = 101), but only few GI, GII.6, and GII.17 cases and no GII.2 cases. The assay validation and our choice of the best threshold to discriminate cases from noncases may therefore be influenced by the disproportionate distribution of the genotypes. Nonetheless, the average assay sensitivity and specificity to identify the correct infecting genotypes is high, particularly considering that the assay was validated using saliva samples from children who were on average 2 years old whose immune system may still be in the developing stage [29].

Other studies have shown that salivary norovirus immunoassays can correctly discriminate infected from uninfected individuals when comparing pre- to postchallenge samples from adult volunteers with PCR-confirmed infection status in a highly controlled setting (challenge study) and that salivary immunoassays can be applied to study asymptomatic norovirus infection in a tropical setting with unknown PCR diagnosis [16, 17, 19]. However, participants in vaccine challenge studies are routinely screened for past infection prior to enrollment and may not be representative of the population response in general, particularly in highly endemic areas. This is, to our knowledge, the first study to systematically validate a salivary norovirus assay for its performance to (1) identify norovirus infection among children with known infection status in a highly endemic setting and (2) discriminate the 5 most common genotypes using saliva samples from children <5 years of age.

Future studies should investigate if a salivary anti-norovirus IgA assay could be used to identify acute infection without the need for repeat sampling. Salivary immunoassays to monitor norovirus infections at the genotype level could be implemented in epidemiological studies in community settings, in outbreak investigations, and in high-risk settings such as schools or healthcare facilities to monitor transmission.

## Notes

***Acknowledgments.*** The authors thank the children and parents who contributed time and samples to this study, as this study would not have been possible without them. We also thank the staff of the MAL-ED Network for their important contributions and Robert Atmar for the provision of VLPs.

***Financial support.*** This work was supported by the Sherrilyn and Ken Fisher Center for Environmental Infectious Diseases, Division of Infectious Diseases of the Johns Hopkins University School of Medicine. Additional funding was obtained from the Bill & Melinda Gates Foundation (grant number 113805); the Foundation for the National Institutes of Health; and the National Institutes of Health Fogarty International Center through joint support of MAL-ED and the Asprey Foundation in Maryland (to K. S.).

***Potential conflicts of interest.*** D. A. G. is founder and Chief Scientific and Strategy Advisor at Salimetrics LLC and SalivaBio LLC. The nature of these relationships is managed by the policies of the committees on conflicts of interest at the Johns Hopkins University School of Medicine and the University of California, Irvine. All other authors report no potential conflicts. All authors have submitted the ICMJE Form for Disclosure of Potential Conflicts of Interest. Conflicts that the editors consider relevant to the content of the manuscript have been disclosed.

Presented in part: Sherrilyn and Ken Fisher Center Infectious Diseases Seminar, Johns Hopkins School of Medicine, Baltimore, Maryland, September 2017.
